# Supplementary material for: Body Weight Changes During Childhood and Predictors of Excessive Body Weight in Adolescence—A Longitudinal Analysis
Source: Nutrients. 2024 Dec 21;16(24):4397. doi: 10.3390/nu16244397 (PMC11676939; doi:10.3390/nu16244397)
Supplement: Supplementary file 1 [file nutrients-16-04397-s001.zip › nutrients-3360483-supplementary.pdf]

KPRT – multivariate logistic regression models

Model 1. Multivariable logistic regression – body weight of 6 y.o. children.

Predictor	OR	95% CI Lower	95% CI Upper	P-value
Good KPRT score (ref)	-	-	-	-
Poor KPRT score	3.20	2.51	4.09	< 0.001
Sex - girls (ref)	-	-	-	-
Sex - boys	1.26	1.00	1.60	0.048

Note: The dependent variable in this analysis is body weight coded so that 0 = did not have overweight or obesity and 1 = have overweight or obesity.

Model 2. Multivariable logistic regression – body weight of 10 y.o. children.

Predictor	OR	95% CI Lower	95% CI Upper	P-value
Good KPRT score (ref)	-	-	-	-
Poor KPRT score	2.58	2.05	3.24	< 0.001
Sex – girls (ref)	-	-	-	-
Sex - boys	1.17	0.93	1.46	0.175

Note: The dependent variable in this analysis is body weight coded so that 0 = did not have overweight or obesity and 1 = have overweight or obesity.

Model 3. Multivariable logistic regression – body weight of 14 y.o. children.

Predictor	OR	95% CI Lower	95% CI Upper	P-value
Good KPRT score (ref)	-	-	-	-
Poor KPRT score	2.65	2.15	3.25	< 0.001
Sex – girls (ref)	-	-	-	-
Sex - boys	1.19	0.97	1.47	0.099

Note: The dependent variable in this analysis is body weight coded so that 0 = did not have overweight or obesity and 1 = have overweight or obesity.
